# Patients who leave Emergency Department without being seen or during treatment in the Lazio Region (Central Italy): Determinants and short term outcomes

**DOI:** 10.1371/journal.pone.0208914

**Published:** 2018-12-12

**Authors:** Francesca Mataloni, Paola Colais, Claudia Galassi, Marina Davoli, Danilo Fusco

**Affiliations:** 1 Department of Epidemiology, Lazio Regional Health Service, Rome, Italy; 2 Unit of Clinical Epidemiology, Città della Salute e della Scienza di Torino University Hospital and CPO Piemonte, Torino, Italy; University of California San Diego, UNITED STATES

## Abstract

**Background and Aims:**

Patients who leave Emergency Department before physician’s visit (LWBS) or during treatment (LDT) represent a useful indicator of the emergency care's quality. The profile of patients LWBS was described: they are generally males, young, with lower urgency triage allocation and longer waiting time. They have a greater risk of ED re-admission compared to discharged patients, but effect on hospitalization and mortality are more controversial. The aims of this study are to identify determinants and adverse short term outcomes for LWBS and LDT patients.

**Methods:**

This is a retrospective cohort study that include all ED visits of LWBS, LDT and discharged patients in 2015 in the Lazio region, Central Italy. Determinants of LWBS or LDT were selected from gender, age, citizenship, residence area, triage category, chronic comorbidities, number of uncompleted ED visit in the previous year, mode of arrival in ED, time-band, day of the week, waiting time and ED crowding, using a multi-level logistic regression. A multivariate logistic regression was used to test if LWBS or LDT have a greater risk of short term adverse outcome compared to discharged patients.

**Results:**

The cohort consists in 835,440 visits in ED, 86.8% subjects visited and discharged, 8.9% subjects are LWBS patients and 4.3% LDT. LWBS and LDT patients are mainly young, males, with a less severe triage, with long waiting times in ED. Moreover, ED crowding and leaving ED before physician’s visit in the previous year are risk factors of self-discharging. LWBS and LDT patients have a higher risk of readmission (LWBS: OR = 4.63, 95%CI 4.5–4.7; OR = 2.89, 95%CI 2.8–2.9; LDT: OR = 3.12, 95%CI 3–3.2; OR = 2.25, 95%CI2.2–2.3 for readmissions within 2 and 7 days respectively) and hospitalization (LWBS: OR = 3.65, 95%CI 3.4–3.9; OR = 2.25, 95%CI 2.1–2.4; LDT: OR = 3.96, 95%CI 3.6–4.3; OR = 2.62, 95%CI 2.4–2.8 for hospitalization within 2 and 7 days respectively). Furthermore, we find a mortality excess of risk for LWBS patients compared to the reference group (OR = 2.56, 95%CI1.6–4.2; OR = 1.7, 95%CI 1.3–2.2 within 2 and 7 days respectively).

**Conclusions:**

Determinants of LWBS confirmed what already known, but LDT patients should be further investigated. There could be adverse health effects for people with LWBS and LDT behaviour. This could be an issue that the Regional Health System should deal with.

## Introduction

Patients who leave the Emergency Department before physician’s visit or treatment represent a useful indicator of the emergency care's quality. In 2012 leaving without being seen (LWBS) rates have been included among the 10 indicators of performance in the national US quality forum [1].

The percentage of LWBS patients is variable between hospitals and countries: (0.84% - 15% in the USA, 1.1% - 10.1% in Australia, 3.26% is the average in England). Lowest rates were observed in Asia: 0.36% in Hong Kong and 0,1% in Taiwan [2–3]. A paper published in 2008 reported increased rates in Spain, compared to the previous analysis conducted 10 years before [4].

Rates of LWBS vary also by type of hospital: lower rates were found for paediatric hospitals, for public hospitals and for teaching hospitals compared to the others [5–11].

Two recent systematic reviews summarized the determinants of leaving the ED without being seen (LWBS) and the health consequences associated to this decision [2–3]. The profile of LWBS patients was described: they are generally males, young, with lower urgency triage allocation and longer waiting times [12–21]. Patients who do not arrive by assisted transport, who arrive during the night and weekend are also at greater risk of LWBS [19–20]. ED crowding is also identified as one of the most important risk factors of patients self-discharging [16, 22–28].

Patients who “did not wait” for medical assistance sought alternate medical care through their personal physician or other EDs [29] and have a higher risk of re-presenting to an ED within 48 hours, compared to those who completed the treatment [19]. Increased risk of mortality within 2 and 7 days was found when models were adjusted for patients, ED visit, temporal and hospital variables. Moreover, lower risks of hospital admission 48 hours after discharge were found. Guttman found no excess of hospital admission and a lower risk of mortality for LWBS patients compared to those discharged who had been waiting less than 1 hour for a medical visit [30]. Similarly, a previous Australian study had found that the 30-day mortality rate was significantly lower among those who did not wait to be seen compared to those who were [11].

Literature on Italian data is scarce; in 2011 a study was conducted to describe demographic and clinical characteristics of patients who left the ED before physician visit in a large teaching hospital in Florence (Azienda Ospedaliero-Universitaria Careggi) [27]. Very low levels of LWBS were found (2%).

In 2015, the percentage of LWBS in Italy was 2.1% and varies from 0.1% (Friuli Venezia Giulia) to 5.9% (Lazio Region) [31].

Furthermore, in Italy there is also a remarkable percentage of patients who leave the ED during physician visit or treatment (LDT) who could represent an interesting phenomenon that should be further explored. The percentage of LDT patients ranges from 0.7% (Lombardia and Emilia Romagna Regions) to 9.1% (Sicilia Region), with a mean national value of 2.6%. The LDT rate in the Lazio Region is equal to 3.18% [31].

The aims of this study are:

1. to identify risk factors associated with patients who arrive at the ED in the Lazio Region in 2015 and
  - leave before the visit or treatment (LWBS);
  - leave the ED during the visit or treatment (LDT);
2. to evaluate adverse short term outcomes (new ED accesses, hospitalizations, mortality) for LWBS and LDT patients compared to those who are visited and discharged.

## Material and methods

### Setting and participants

This is a retrospective cohort study that includes all ED visits from January 1, 2015 to December 31, 2015 in the Lazio region, Central Italy. The units of analysis are ED visits. For patients who accessed the ED more than one time during the year, each visit was counted as a separate data point. All visits of patients who left ED without being seen (LWBS) or who left ED during treatment (LDT) were selected and compared to visits of patients who were discharged at home after ED visit. Therefore, ED visits of patients who at discharge were hospitalized, dead, transferred and who have refused hospitalization were not included in the study, because outcomes under study were readmission, hospitalization and mortality. Moreover, we excluded visits of patients who were not residents of the Lazio region because the data source consisted in the Regional Health Information System. Patients younger than 18 years were also excluded because their decisions about medical care are likely made by their parents. Lastly, these categories were also excluded: red or missing triage code assignments; patients with symptoms of shock or coma; ED visits lasting longer than 24 hours because considered unreliable; and visits with a diagnosis involving pregnancy or delivery (ICD-9-CM codes V27, 650, 640–676 with 1 or 2 as the fifth number). In case of multiple visits by the same patient within 7 days, only the first ED visit was considered as the “index-visit”. However, we included readmissions to ED within 2 days and within 7 days because these were considered as possible short term outcomes.

### Data sources

We collected data from the Healthcare Emergency Information System (HEIS), the Hospital Information System (HIS) and the Regional Tax Registry.

The HEIS was used to enrol our cohort. This database includes all visits occurring in Emergency Departments of the Lazio region and collects: patient demographics, admission information, visit and discharge dates and hours, ICD-9-CM diagnosis at discharge, reported symptoms on arrival, status at discharge (e.g., dead, hospitalized, or discharged at home) and triage score (from white to red).

The HIS is an integrated information system designed to collect clinical and administrative information regarding hospital admissions for each patient discharged from public and private hospitals of the Region.

The Regional Tax Registry was used to gather fiscal information and to verify the life status for every patient.

The cohort of ED visits was linked to the HIS to define patients’ comorbidities and hospitalization outcomes and to the Regional Tax Registry to establish the vital status, available even for people who died outside of hospital. Data from different information systems were merged by a deterministic record linkage procedure based on anonymous identification codes, unique for patients in the Region.

### Statistical analysis

In the first analysis outcome under study are LWBS and LDT and we identified which determinants could cause this behaviour. Several risk factors were considered:

- Demographic characteristics: gender, age, citizenship (foreign vs Italian) and residence area (city of Rome, province of Rome vs others provinces);
- Health status and attitude of self-discharging: triage category (less urgent vs more urgent), hospitalizations during the previous 5 years and number of uncompleted ED visit in the previous year (1, >1 vs 0);
- Visit characteristics: mode of arrival in ED (non-assisted arrival vs ambulance), time-band (8:00–15:00, 16:00–23:00 and 00:00–7:00) and day of the week (weekend/holiday vs weekday);
- Possible ED organization problems: waiting time (from less than 1 hour to more than 6 hours), ED crowding (yes vs no).

All risk factors were identified from the Regional Health Information System: HEIS was used to collect all information related to the patients’ characteristics and ED visit; HIS was used to identify patients with chronic comorbidities or a post-acute status: hospitalization (in the previous 5 years from the index visit) for malignant cancer, diabetes, obesity, haematological diseases, hypertension, ischemic heart disease, heart failure, arrhythmias, cerebrovascular diseases, vascular diseases, COPD, chronic diseases (liver, pancreas, intestine), anaemia and coagulopathy and others (ICD-9-CM codes in S1 Table) were taken into account.

Information related to ED crowding was defined by counting the number of patients present in the ED at the time of each new arrival. If this number resulted in greater than the 75° percentile of the distribution of patients generally present in the ED at that time-band, the ED was defined as crowded [32].

Determinants of LWBS or LDT were analysed using a multi-level logistic regression considering ED visits as the first level unit and the ED as the second one.

In the second analysis we tested if LWBS or LDT have a greater risk of short term adverse outcome compared to discharged patients. Outcome measures were defined as follows:

- Readmission to ED within 2 days and 7 days;
- Hospitalization within 2 days and 7 days;
- Mortality within 2 and 7 days.

Only ordinary hospitalizations of stays greater than one day were considered outcomes of interest for our analysis. Moreover, we excluded outcomes of readmissions or hospitalizations with a diagnosis of pregnancy and delivery (ICD-9-CM codes: V27, 650, 640–676 with 1 or 2 as the fifth number).

Multivariate logistic regression models (ORs and 95% CIs) were performed, adjusting for gender, age, ED crowding and triage. These confounders were identified using a directed acyclic graph (DAG) (S1 Fig).

The SAS (SAS Institute Inc., North Carolina) software programme was used for statistical analyses.

## Results

In the Lazio region, there are 50 EDs, 22 of which are in Rome; we excluded two high-specialized ED from our study (one dental ED and one ophthalmic ED). Therefore, we considered a cohort of visits to 48 EDs with a large range of LWBS (from 0% to 24.3%, with a mean rate of 8.9%) and of LDT (from 0.2% to 16.8%, with a mean rate of 4.30%). Rates of LWBS and LDT are generally lower for low volume hospitals (LWBS: 5.9% LDT: 3%), nevertheless some small EDs have rates that exceed the regional mean (LWBS from 0% to 11.5%; LDT from 0.2% to 6.2%). Teaching hospitals had lower rates of self-discharging patients (6% of LWBS and 3.4% of LDT).

**Fig 1:**
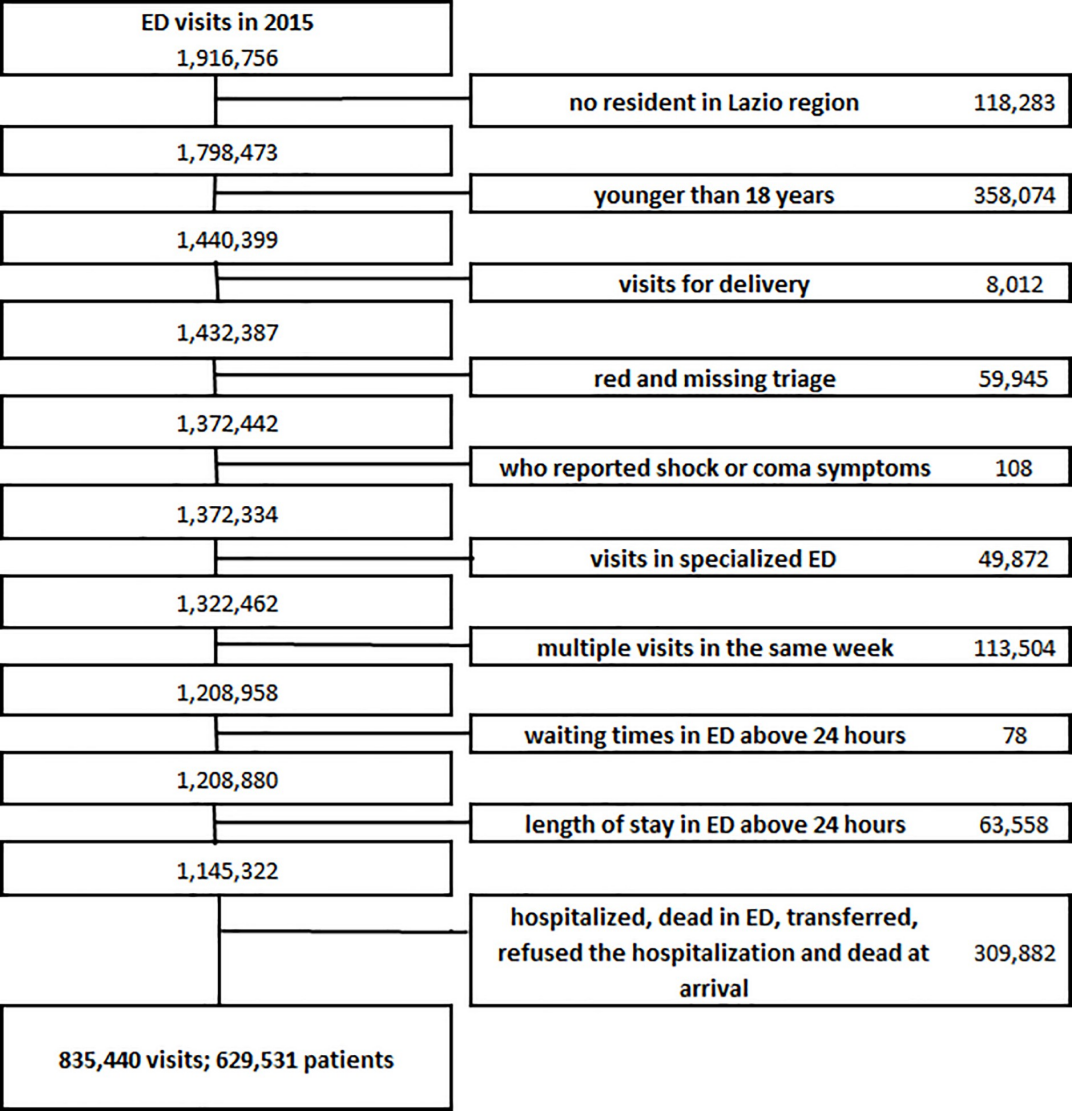
Summary of the cohort enrolment—exclusion criteria.

A total of 1,916,756 ED visits were provided in the Lazio region during 2015; after the application of exclusion criteria, the cohort consisted in 835,440 visits corresponding to 629,531 patients (Fig 1). In fact 75.4% of the visits enrolled in the cohort correspond to people with one access to ED in 2015, 14.9% to people who had 2 accesses in the same year and 9.8% to people with more than 2 visits. The majority of patients that left the ED without being seen or during the treatment did so only one time during the study period.

The main characteristics of the cohort are described in Table 1. The cohort consists of 724,961 (86.8%) subjects visited and discharged, 74,523 (8.9%) subjects that left without being seen and 35,956 (4.3%) subjects that left during treatment. Of note, 52% are female, but patients who leave the ED are more frequently male (LWBS 52.1%, LDT 51%). LWBS and LDT patients are generally younger with the percentage of patients older than 70 years lower in this group compared to discharged patients (13.2% and 13.6% vs 18.1%). 82.1% of the cohort is Italian versus foreign. 45.6% of the cohort visits refer to subjects residing in Rome, while 21.9% of LWBS and 31.4% of LDT comes from provinces outside of Rome.

**Table 1:** Characteristics of the overall study cohort (Total ED visits), and separately for visits of subjects visited and discharged, those who left the ED without being seen (LWBS) or during the treatment (LDT).

Total		Total		Discharged		LWBS		LDT	
		n	%	n	%	n	%	n	%
		835,440	100	724,961	100	74,523	100	35,956	100
**Gender**	*Male*	400,013	47.9	342,823	47.3	38,843	52.1	18,347	51.0
	*Female*	435,427	52.1	382,138	52.7	35,680	47.9	17,609	49.0
**Age**	*19–30*	147,113	17.6	124,262	17.1	15,537	20.8	7,314	20.3
	*30–40*	159,897	19.1	137,145	18.9	15,114	20.3	7,638	21.2
	*40–50*	159,619	19.1	137,113	18.9	15,468	20.8	7,038	19.6
	*50–60*	127,659	15.3	111,362	15.4	11,131	14.9	5,166	14.4
	*60–70*	95,393	11.4	84,015	11.6	7,470	10.0	3,908	10.9
	*70+*	145,759	17.4	131,064	18.1	9,803	13.2	4,892	13.6
**citizenship**	*Italian*	685,530	82.1	596,440	82.3	60,049	80.6	29,041	80.8
	*Foreign*	149,910	17.9	128,521	17.7	14,474	19.4	6,915	19.2
**Residence**	*Rome*	388,170	46.5	333,525	46.0	37,860	50.8	16,785	46.7
	*Rome province*	216,053	25.9	187,840	25.9	20,348	27.3	7,865	21.9
	*Other provinces*	231,217	27.7	203,596	28.1	16,315	21.9	11,306	31.4
**Previous (5 years) hospitalizations**	*No*	625,315	74.8	538,934	74.3	59,028	79.2	27,353	76.1
	*Yes*	210,125	25.2	186,027	25.7	15,495	20.8	8,603	23.9
**Triage**	*Yellow*	179,569	21.5	167,839	23.2	6,275	8.4	5,455	15.2
	*Green*	614,751	73.6	529,068	73.0	58,944	79.1	26,739	74.4
	*White*	41,120	4.9	28,054	3.9	9,304	12.5	3,762	10.5
**Number of uncompleted ED visit in the previous year**	*0* *1* *>1*	773,571	92.6	677,191	93.4	65,887	88.4	30,493	84.8
		47,861	5.7	38,816	5.4	5,706	7.7	3,339	9.3
		14,008	1.7	8,954	1.2	2,930	3.9	2,124	5.9
**Symptoms**	*Nervous system*	12,364	1.5	9,996	1.4	1,274	1.7	1,094	3.0
	*Abdominal pain*	56,485	6.8	49,629	6.8	4,467	6.0	2,389	6.6
	*Chest pain*	25,302	3.0	22,490	3.1	1,680	2.3	1,132	3.1
	*Dyspnea*	10,576	1.3	9,165	1.3	896	1.2	515	1.4
	*Nontraumatic hemorrhage*	9,574	1.1	8,520	1.2	623	0.8	431	1.2
	*Trauma or burns*	276,291	33.1	251,823	34.7	17,965	24.1	6,503	18.1
	*Poisoning*, *allergic reaction*	7,277	0.9	5,865	0.8	976	1.3	436	1.2
	*Fever*	9,650	1.2	7,942	1.1	1,189	1.6	519	1.4
	*Heart rhythm*, *hypertension*	16,827	2.0	14,916	2.1	1,298	1.7	613	1.7
	*Eye*, *ear*, *nose*, *skin*	45,351	5.4	37,983	5.2	3,507	4.7	3,861	10.7
	*Genitourinary and obstetric*	57,126	6.8	52,128	7.2	1,769	2.4	3,229	9.0
	*Others*	307,671	36.8	253,883	35.0	38,801	52.1	14,987	41.7
	*Administrative issue*	946	0.1	621	0.1	78	0.1	247	0.7
**Symptoms duration (hours)**	*<6*	404,574	48.4	356,710	49.2	31,860	42.8	16,004	44.5
** **	*>6*	430,866	51.6	368,251	50.8	42,663	57.2	19,952	55.5
**Mode of arrival**	*ambulance*	114,414	13.7	100,571	13.9	8,512	11.4	5,331	14.8
	*non-assisted arrival*	721,026	86.3	624,390	86.1	66,011	88.6	30,625	85.2
**Time band**	*8*:*00–15*:*00*	464,018	55.5	405,538	55.9	38,044	51.1	20,436	56.8
	*16*:*00–23*:*00*	288,404	34.5	245,456	33.9	30,949	41.5	11,999	33.4
	*00*:*00–7*:*00*	83,018	9.9	73,967	10.2	5,530	7.4	3,521	9.8
**Week-end/Holiday**	*No*	588,734	70.5	510,203	70.4	52,279	70.2	26,252	73.0
	*Yes*	246,706	29.5	214,758	29.6	22,244	29.8	9,704	27.0
**Season**	*Winter*	202,216	24.2	173,188	23.9	19,828	26.6	9,200	25.6
	*Spring*	216,631	25.9	187,943	25.9	19,112	25.6	9,576	26.6
	*Summer*	221,816	26.6	192,833	26.6	19,938	26.8	9,045	25.2
	*Autumn*	194,777	23.3	170,997	23.6	15,645	21.0	8,135	22.6
**Waiting time (hours)**	*<1*	518,288	62.0	484,695	66.9	13,301	17.8	20,292	56.4
	*1–2*	139,855	16.7	121,185	16.7	13,289	17.8	5,381	15.0
	2–3	69,573	8.3	54,411	7.5	11,873	15.9	3,289	9.1
	3–4	40,478	4.8	28,554	3.9	9,658	13.0	2,266	6.3
	4–5	24,934	3.0	15,978	2.2	7,447	10.0	1,509	4.2
	5–6	15,640	1.9	8,988	1.2	5,596	7.5	1,056	2.9
	>6	26,672	3.2	11,150	1.5	13,359	17.9	2,163	6.0
**Crowding**	*No*	643,750	77.1	568,711	78.4	49,075	65.9	25,964	72.2
	*Yes*	191,690	22.9	156,250	21.6	25,448	34.1	9,992	27.8

The percentage of visits who have had hospitalizations in the previous 5 years for chronic diseases, or for diseases that could cause a post-acute status is 25.7% in discharged patients, 20.8% in LWBS and 23.9% in LDT. As expected, we saw differences in triage codes: LWBS and LDT have a lower percentage of yellow triage (8.4% and 15.2% respectively) compared to patients discharged (23.2%) The percentage of patients who have had an incomplete ED visit in the previous year is higher for LWBS (11.6%) and LDT (15.2%) patients, compared to discharged ones (6.6%). 36.8% of the study cohort declared symptoms at arrival in ED recorded as “other” and 33.1% declared symptoms of trauma or burns.

More than 13% were transport-assisted to the ED. The majority of visits (55.5%) were in the morning (8:00–15:00); there were no important differences between distribution of week-day, time-band and season between patients discharged and LWBS or LDT.

22.9% of the total cohort arrived in a crowded ED, this percentage goes up to 34.1% and 27.8% among LWBS and LDT patients respectively. 62% of the total cohort waited less than 1 hour for the physician visit and 3.2% more than 6 hours. These wait times are quite different when broken down into groups: (LWBS: 17.8% <1 hour and 17.9% >6 hours; LDT: 56.4% <1 hour and 6% >6 hours) compared to discharged ones (66.9% <1 hour and 1.5% >6 hours).

In Table 2 risk factors for LWBS are shown. The risk of leaving the ED without being seen decreases with age (OR = 0.93 for 10 years increase), is lower in females (OR = 0.86), for patients with previous hospitalizations for chronic comorbidities or for disease that could cause a post-acute status (OR = 0.94) and for patients with foreign citizenship (OR = 0.94). The risk of LWBS is higher for non-assisted arrival (OR = 1.22), for patients who arrived during the afternoon and the night (OR = 1.46 and OR = 1.16), during week-end or holidays (OR = 1.12) and in a crowded ED (OR = 1.30). This risk increases with decreasing urgency of the triage category (OR = 2.05 for green triage, OR = 7.01 for white triage, compared to yellow ones) and with increasing wait times (OR = 32.31 >6 hours compared to those who wait <1 hour). The risk of LWBS is lower for resident in the province of Rome compared to those living in the rest of the region (OR = 0.90). The tendency to leave ED is higher for patients who have already done it once (OR = 1.45) or more than once in the previous year (OR = 3.26).

**Table 2:** Risk factors of leaving without being seen (LWBS)—results from a multi-level logistic regression model.

Risk factor		n	Crude OR	95% CI		Adj OR	95% CI		p value
				Low	Up		Low	Up	
Age (10 years increase)		.	0.92	0.91	0.92	0.93	0.93	0.94	< .0001
Gender (Female vs Male)		417,818	0.80	0.79	0.81	0.86	0.84	0.87	< .0001
Citizenship (foreign vs italian)		142,995	1.39	1.36	1.42	0.94	0.91	0.96	< .0001
Residence area	Roma	371,385	1.05	1.01	1.10	1.03	0.98	1.07	0.247
	Rome province	208,188	0.88	0.85	0.92	0.90	0.86	0.94	< .0001
	Others province	219,911	1.00	.	.	1.00	.	.	.
Previous (5 years) hospitalizations		201,522	0.77	0.75	0.78	0.94	0.92	0.96	< .0001
Triage	Yellow	174,114	1.00	.	.	1.00	.	.	.
	Green	588,012	3.30	3.22	3.40	2.05	1.99	2.11	< .0001
	White	37,358	10.91	10.51	11.33	7.01	6.72	7.31	< .0001
Number of uncompleted ED visit in the previous year	0	743,078	1.00	.	.	1.00	.	.	.
	1	44,522	1.46	1.42	1.51	1.45	1.40	1.50	< .0001
	>1	11,884	3.22	3.08	3.37	3.26	3.10	3.44	< .0001
Mode of arrival (non-assisted arrival vs ambulance)		690,401	1.30	1.27	1.34	1.22	1.18	1.25	< .0001
Time band	8:00–16:00	443,582	1.00	.	.	1.00	.	.	.
	17:00–24:00	276,405	1.34	1.32	1.36	1.46	1.44	1.49	< .0001
	1:00–7:00	79,497	0.75	0.73	0.77	1.16	1.12	1.20	< .0001
Day (Week-end/Holiday vs weekday)		237,002	1.00	0.99	1.02	1.12	1.10	1.14	< .0001
Waiting time (hours)	<1	497,996	1.00	.	.	1.00	.	.	.
	1–2	134,474	3.79	3.70	3.89	3.57	3.48	3.66	< .0001
	2–3	66,284	7.48	7.28	7.68	6.83	6.64	7.02	< .0001
	3–4	38,212	11.27	10.94	11.61	10.12	9.81	10.44	< .0001
	4–5	23,425	15.20	14.69	15.72	13.52	13.06	14.01	< .0001
	5–6	14,584	19.82	19.06	20.62	17.47	16.77	18.20	< .0001
	>6	24,509	36.81	35.63	38.03	32.31	31.21	33.44	< .0001
ED crowding (Yes vs No)		181,698	1.88	1.85	1.91	1.30	1.28	1.33	< .0001

In Table 3 the results of the multi-level model for LDT patients are shown. As for LWBS, the risk of leaving the ED during treatment decreases with age (OR = 0.94 for 10 years increase), is lower for females (OR = 0.90). Also, this risk is higher in crowded ED (OR = 1.33), increases with decreasing of triage category (OR = 1.85 for green triage, OR = 4.96 for white triage, compared to yellow ones) and with increasing wait times, although with a lower OR compared to LWBS (OR = 1.86 for >5 hours compared to those who wait <1 hour). The tendency to leave ED during treatment is higher for patients who have already left the ED once in the previous year (OR = 1.72) or more than once (OR = 4.68). Contrary to the results for LWBS, the risk of LDT was not associated with a previous hospitalization and with non-assisted arrival, is higher for foreign citizenship (OR = 1.14) and for patients residing in Rome (OR = 1.07) is lower for patients who arrived during the night (OR = 0.89) and during week-end or holidays (OR = 0.92).

**Table 3:** Risk factors of leaving during treatment (LDT)—results from a multi-level logistic regression model.

Risk factor		n	Crude OR	95% CI		Adj OR	95% CI		p value
				Low	Up		Low	Up	
Age (10 years increase)		.	0.92	0.92	0.93	0.94	0.93	0.95	< .0001
Gender (Female vs Male)		399,747	0.89	0.87	0.91	0.90	0.88	0.92	< .0001
Citizenship (foreign vs italian)		135,436	1.38	1.34	1.42	1.14	1.10	1.17	< .0001
Residence area	Rome	350,310	1.08	1.02	1.14	1.07	1.01	1.13	0.030
	Rome province	195,705	1.00	0.95	1.06	1.00	0.95	1.06	0.960
	Others province	214,902	1.00			1.00	.	.	.
Previous (5 years) hospitalizations		194,630	0.88	0.86	0.90	0.99	0.97	1.02	0.728
Triage	Yellow	173,294	1.00			1.00	.	.	.
	Green	555,807	1.73	1.68	1.79	1.85	1.79	1.91	< .0001
	White	31,816	4.53	4.32	4.75	4.96	4.72	5.22	< .0001
Number of uncompleted ED visit in the previous year	0	707,684	1.00			1.00	.	.	.
	1	42,155	1.79	1.72	1.86	1.72	1.65	1.79	< .0001
	>1	11,078	4.90	4.65	5.16	4.68	4.44	4.94	< .0001
Mode of arrival (non-assisted arrival vs ambulance)		655,015	1.06	1.03	1.09	0.99	0.95	1.02	0.373
Time band	8:00–16:00	425,974	1.00			1.00	.	.	.
	17:00–24:00	257,455	1.00	0.97	1.02	1.02	0.99	1.04	0.150
	1:00–7:00	77,488	0.91	0.88	0.94	0.89	0.85	0.92	< .0001
Day (Week-end/Holiday vs weekday)		224,462	0.88	0.86	0.90	0.92	0.90	0.94	< .0001
Waiting time (hours)	<1	504,987	1.00			1.00	.	.	.
	1–2	126,566	1.07	1.03	1.11	1.14	1.10	1.18	< .0001
	2–3	57,700	1.18	1.14	1.23	1.33	1.28	1.38	< .0001
	3–4	30,820	1.31	1.25	1.36	1.51	1.45	1.57	< .0001
	4–5	17,487	1.41	1.35	1.48	1.67	1.59	1.75	< .0001
	>5	10,044	1.47	1.42	1.52	1.86	1.79	1.94	< .0001
ED crowding		166,242	1.37	1.34	1.40	1.33	1.30	1.36	< .0001

Shown in Table 4 are the risks of readmission to ED, hospitalization and mortality within 2 and 7 days, for LWBS and LDT patients compared to those visited and discharged.

**Table 4:** Associations between leaving ED (LWBS and LDT) and readmission, hospitalization and mortality within 2 and 7 days: Crude Rate (Rate*100), Crude Odds Ratio (Crude OR).

	**Outcome**						
	**Readmission within 2 days**						
	**n**	Rate*100	**95% CI**	**Crude OR**	**95% CI**	**Adj OR**	**95% CI**
**Total ED cohort**	46,470	5.56	5.51–5.61	**-**	**-**	**-**	**-**
**Discahrged**	30,538	4.21	4.17–4.26	1	**-**	1	-
**LWBS**	11,763	15.78	15.52–16.05	4.26	4.17–4.36	4.63	4.53–4.75
**LDT**	4,169	11.59	11.26–11.93	2.98	2.88–3.09	3.12	3.02–3.23
	**Readmission within 7 days**						
	**n**	Rate*100	**95% CI**	**Crude OR**	**95% CI**	**Adj OR**	**95% CI**
**Total ED cohort**	84,110	10.07	10.00–10.13	-	**-**	**-**	**-**
**Discahrged**	62,990	8.69	8.62–8.75	1	**-**	1	**-**
**LWBS**	15,035	20.17	19.89–20.46	2.66	2.60–2.71	2.89	2.84–2.95
**LDT**	6,085	16.92	16.54–17.31	2.14	2.08–2.20	2.25	2.19–2.32
	**Hospitalization within 2 days**						
	**n**	Rate*100	**95% CI**	**Crude OR**	**95% CI**	**Adj OR**	**95% CI**
**Total ED cohort**	4,773	0.57	0.56–0.59	**-**	**-**	**-**	**-**
**Discahrged**	3,316	0.46	0.44–0.47	1	**-**	1	-
**LWBS**	923	1.24	1.16–1.32	2.73	2.54–2.94	3.65	3.38–3.94
**LDT**	534	1.49	1.36–1.61	3.28	2.99–3.60	3.96	3.60–4.34
	**Hospitalization within 7 days**						
	**n**	Rate*100	**95% CI**	**Crude OR**	**95% CI**	**Adj OR**	**95% CI**
**Total ED cohort**	13,131	1.57	1.55–1.60	-	**-**	**-**	**-**
**Discahrged**	10,299	1.42	1.39–1.45	1	**-**	1	**-**
**LWBS**	1,758	2.36	2.25–2.47	1.68	1.59–1.76	2.25	2.13–2.37
**LDT**	1,074	2.99	2.81–3.16	2.14	2.00–2.28	2.62	2.45–2.79
	**Mortality within 2 days**						
	**n**	Rate*100	**95% CI**	**Crude OR**	**95% CI**	**Adj OR**	**95% CI**
**Total ED cohort**	165	0.02	0.016–0.023	**-**	**-**	**-**	**-**
**Discahrged**	142	0.02	0.016–0.023	1	**-**	1	-
**LWBS**	19	0.03	0.01–0.04	1.30	0.81–2.1	2.56	1.56–4.19
**LDT**	4	0.01	0.00–0.02	0.57	0.21–1.53	0.95	0.35–2.57
	**Mortality within 7 days**						
	**n**	Rate*100	**95% CI**	**Crude OR**	**95% CI**	**Adj OR**	**95% CI**
**Total ED cohort**	775	0.09	0.09–0.10	-	**-**	**-**	**-**
**Discahrged**	680	0.09	0.09–0.10	1	**-**	1	**-**
**LWBS**	65	0.09	0.07–0.11	0.93	0.72–1.2	1.70	1.31–2.21
**LDT**	30	0.08	0.05–0.11	0.89	0.62–1.28	1.41	0.98–2.04

Rates of readmission to ED within 2 and 7 days from the index ED visit are equal to 4.2% and 8.7% for discharged patients; higher for LWBS and LDT patients (15.8% and 11.6% within 2 days and 20.2% and 16.9% within 7 days) compared to discharged patients. The greater risk of readmission for LWBS and LDT patients, was also evident from logistic regression results (LWBS: OR = 4.63, 95%CI 4.53–4.75; OR = 2.89, 95%CI 2.84–2.95 for readmissions within 2 and 7 days respectively; LDT: OR = 3.12, 95%CI 3.02–3.23; OR = 2.25, 95%CI 2.19–2.32 for readmissions within 2 and 7 days respectively).

Hospitalization rates within 2 or 7 days from the index ED visit are lower than readmissions; 0.46% of patients discharged had a subsequent hospitalization within 2 days and 1.4% after 7 days from index visit to ED. LWBS and LDT patients have higher rates of hospitalization (> 1.2% within 2 days and > 2.4% within 7 days) and was confirmed in our regression models: (OR = 3.65, 95%CI 3.38–3.94; OR = 2.25, 95%CI 2.13–2.37 within 2 and 7 days respectively; OR = 3.96, 95%CI 3.60–4.34; OR = 2.62, 95%CI 2.45–2.79 within 2 and 7 days respectively).

A greater risk of mortality within 2 and 7 days was found for LWBS patients (adjOR = 2.56, 95%CI 1.56–4.19 and adjOR = 1.70, 95%CI 1.31–2.21 respectively). When the analysis was adjusted for gender, age, crowding and triage, a suggestion of increasing risk of mortality within 7 days was found also for LDT patients (adjOR = 1.41, 95%CI 0.98–2.04).

## Discussion

In this study we have analysed risk factors and subsequent health outcomes of patients that did not conclude their care pathway in ED and decided to leave before or during physician visit (LWBS and LDT). The literature is rich with studies on the determinants of LWBS and our results confirmed what is already known about it. LWBS patients are mainly young, males, with a less severe triage, without chronic comorbidities and, particularly, with long waiting times in ED. Moreover, ED crowding and leaving ED before physician’s visit in the previous year are risk factors of LWBS. In this study determinants of LDT have been also analysed; contrary to the results for LWBS, the risk of LDT is not associated with a previous hospitalization and with non-assisted arrival, is lower for patients who arrived during the night (OR = 0.89) and during week-end or holidays (OR = 0.92) and is higher for patients residing in the city of Rome (OR = 1.07) compared to the rest of the Region. Our results are comparable to previous studies regarding LWBS determinants [2–3, 12–28, 33]. LDT analysis showed different characteristics compared to LWBS and represent a new result. LDT patients are a particular group of patients with heterogeneous characteristics; it is difficult to hypothesize the reasons for their behaviour. For this reason this group of patients should be further investigated.

With regard to successive health outcomes, LWBS and LDT patients have a higher risk of readmission and hospitalization within 2 and 7 days compared to discharged patients. The risk of readmission is higher for LWBS patients and the risk of hospitalization is greater for LDT patients. Furthermore, we find a mortality excess of risk for LWBS patients (but not for LDT patients) compared to the reference group.

Results on readmission are supported by previous literature. A study in 2003 reported a higher percentage of recurrence to medical care for LWBS patients compared to those who had concluded their access [34]. In agreement with our result, two previous North American surveys found that LWBS patients reported a worse condition one or two weeks after the access compared to patients who had received treatment in the ED, more than a quarter of these patients returned to the ED within 14 days, and 4% required hospitalization [6, 12]. A more recent study conducted in Australia found higher risk of readmission for LWBS patients but lower risk of hospitalization [19].

Results on mortality risk are more controversial: Hall et al. found lower mortality rates (within 30 days) among those who did not wait for the physician (0.14%), compared to those seen (0.20%, p < 0.001) [11]; Guttman et al. found lower risk of death within 7 days for LWBS patients compared to discharged patients who had waited less than 1 hour. [30]; Tropea et al. found an indication of an excess in mortality risk within 2 and 7 days for LWBS patients, but only when the analysis was adjusted for confounders [19]. In our study the excess of mortality for LWBS patients is more evident; the discrepancy with the literature could depend on several factors, including differences in cohort selection; in fact, in the studies by Tropea, Hall and Guttman, no exclusion for age was made, while we excluded patients younger than 18 years. Further analyses could evaluate the potential excess of mortality for LWBS using less restrictive criteria in the selection of the cohort.

For the evaluation of subsequent health outcomes, we used as the reference group only subjects that arrived at the ED, were seen by the physician, and then discharged, following the approach used in other published studies [11,30,19]. Only in the study of Hall et al [11], data for LWBS were also compared with data of ED patients overall, including those hospitalized where applicable, and the analysis confirmed lower 30-days mortality rates for LWBS (0.14%) compared to all patients seen in the ED (1.28%). In a sensitivity analysis we examined the risk of mortality (at 2 and 7 days) for the group of patients that arrived to ED and was subsequently hospitalized, in comparison to discharged patients: as expected their risk of mortality was higher (mortality within 2 days OR = 10.15, 95%CI 8.42–12.24; mortality within 7 days OR = 7.48, 95%CI 6.84–8.18) and substantially higher compared to the risks observed for LWBS and LDT patients.

The databases used in the present study did not include the causes of death; in order to better evaluate the subsequent health outcomes we examined the principal diagnosis of hospitalization within 7 days from the index visit for discharged, LWBS and LDT patients. More than 15% of the hospitalizations for discharged patients were due to diseases of the digestive system (ICD-9-CM codes: 520–579; cholelithiasis and intestinal obstruction without mention of hernia in particular), more than 14% was due to diseases of the circulatory system (ICD-9-CM codes: 390–459, cardiac dysrhythmias and heart failure in particular). LWBS and LDT patients hospitalized 7 days from the first visit in ED reported diagnosis of diseases of the circulatory system (14%) and diseases of digestive system (13%); 12% of them reported a principal diagnosis of mental disorders (ICD-9-CM codes: 290–319). Interestingly, Hall et al [11] report that among the groups of patients with the highest rates of “did not wait” (DNW) presentations there were some with social/behavioural problems.

Our study is based on a large dataset (835,440 visits in ED) and is the largest one done in Italy as it includes almost all regional EDs in the Lazio region. However, the generalizability of our results to other Italian regions could be limited by possible differences in the organization of health care services, as well as by regional differences in the proportion of LWBS and LDT [31]; our results support the conduction of similar studies in other regional settings.

Some previous studies evaluated determinants of ED leaving by using surveys [10, 29, 33–34]. In our study, information on LWBS and LDT determinants were derived from administrative data. For this reason, it is not possible to evaluate the quality and completeness of the data. Furthermore, data on medical staff and shift during the day for each ED are not available. The use of administrative data could generate a bias in the cohort selection and in the definition of its characteristics. In addition, the unavailability of some information generate unmeasured confounding that is impossible to quantify or to control. In spite of this, several information from HIS were used, to better characterize patients and to identify risk factors associated to LWBS and LDT: patients’ characteristics (gender, age, citizenship, residence), their health conditions (previous comorbidities, triage score), their tendencies to leave the ED (number of uncompleted ED visit in the previous year) and characteristics of ED visit (waiting time, crowding, mode of arrival, time band and weekend/holiday vs. weekday). The models on health outcomes were adjusted for gender and age, triage score and ED condition at their arrival (ED crowding), but we cannot exclude the possibility of residual and unmeasured confounding that may distort the results.

As shown, there could be adverse health effects for people with LWBS and LDT behaviour. This is an issue that the Regional Health System might want to address, and to develop possible solutions in the management of the Emergency care, outpatient and Primary care services.

In conclusion this study has identified the principal characteristics of LWBS and LDT patients in the Lazio Region, highlighting the possible health consequences of this decision; and could be used to improve the quality of ED care in Italy.

## Supporting information

S1 Fig DAG of the association between ED self-discharging and re-admission, hospitalization and mortality. (TIF)

S1 Table ICD9-CM codes for identification of chronic comorbidities or diagnosis that could cause a post-acute status. (XLS)
