# Supplementary figures and images for: Patients who leave Emergency Department without being seen or during treatment in the Lazio Region (Central Italy): Determinants and short term outcomes
Source: PLoS One. 2018 Dec 12;13(12):e0208914. doi: 10.1371/journal.pone.0208914 (PMC6291150; doi:10.1371/journal.pone.0208914)

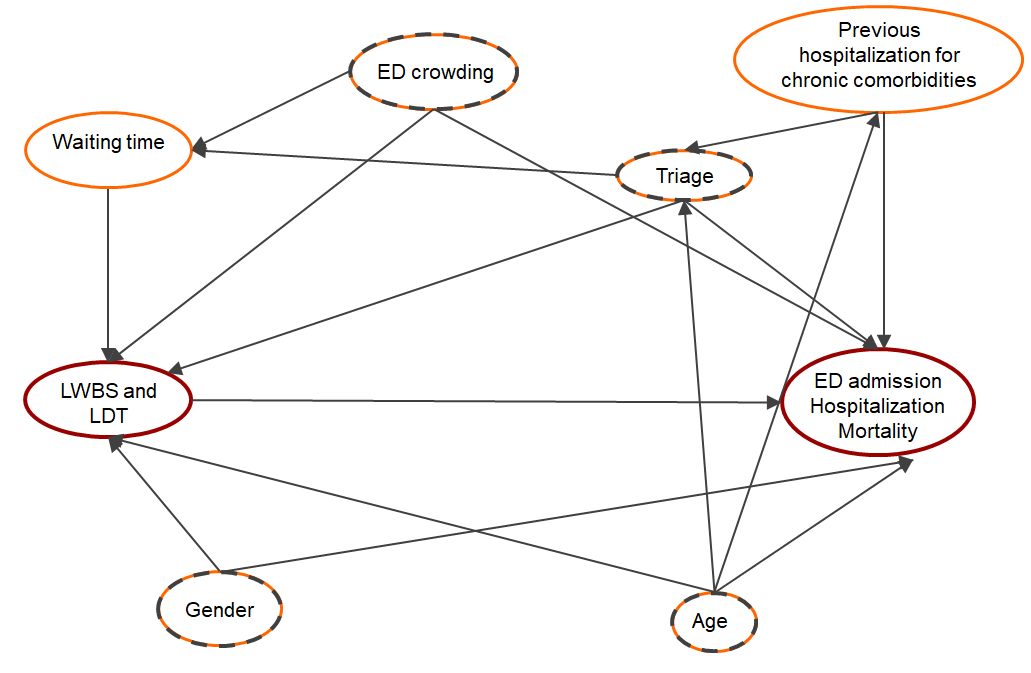

Supplement: S1 Fig — (TIF) [file pone.0208914.s001.tif]
